# Bilateral branch retinal vein occlusion following the diagnosis of mild coronavirus disease

**DOI:** 10.5935/0004-2749.20230017

**Published:** 2023

**Authors:** Buğra Karasu, Enes Kesim

**Affiliations:** 1 Tuzla State Hospital, Department of Ophthalmology, Istanbul, Turkey.

**Keywords:** Retinal vein occlusion, Coronavirus infections, COVID-19, SARS-CoV-2, Oclusão da veia retiniana, Infecções por coronavírus, COVID-19, SARS-CoV-2

## Abstract

The aim of this case report is to present the case of a patient diagnosed as having coronavirus disease (COVID-19) who developed branch retinal vein occlusion in both eyes at different time points. A 48-year-old male patient was admitted to our hospital with symptoms of mild COVID-19 and was diagnosed as having severe acute respiratory syndrome coronavirus 2 (SARS-CoV-2) infection after polymerase chain reaction testing. Two months after the diagnosis, branch retinal vein occlusion was found in his left eye on fundoscopic examination, with a visual acuity of 20/100. In the third month of therapy, the same symptoms developed in the right eye and was diagnosed as branch retinal vein occlusion. The visual acuity was 10/100 in his right eye, which increased to 40/100 in the right eye and 30/100 in the left eye after treatment. The development of branch retinal vein occlusion can be observed during the mild stage of COVID-19, which triggers viral microangiopathy and hypercoagulation. Physicians should be strictly vigilant for retinal assessment in patients with vision loss due to a mild history of COVID-19.

## Abstract (pt)

Este relato apresenta o caso de um paciente com diagnóstico de doença por coronavírus de 2019 (COVID-19) que, em diferentes momentos, desenvolveu oclusão de ramos da veia retiniana em ambos os olhos. Um paciente do sexo masculino de 48 anos foi admitido no hospital com sintomas de COVID-19 leve e a presença do coronavírus da síndrome respiratória aguda grave 2 (SARS-CoV-2) foi constatada através de um teste de reação em cadeia de polimerase. Dois meses após o diagnóstico, uma fundoscopia revelou a oclusão de um ramo da veia retiniana em seu olho esquerdo e constatou-se uma acuidade visual de 20/100 no mesmo olho. No terceiro mês de tratamento, os mesmos sintomas desenvolveram-se no olho direito e foi diagnosticada a oclusão de um ramo da veia retiniana. Constatou-se uma acuidade de 10/100 no olho direito. Após o tratamento, a acuidade visual aumentou para 40/100 no olho direito e 30/100 no olho esquerdo. O desenvolvimento de oclusões de ramos da veia retiniana pode ser observado em casos leves de COVID-19, que desencadeia microangiopatia viral e hipercoagulação. Os médicos devem estar altamente vigilantes para uma avaliação da retina em pacientes com perda de visão devido a uma história de COVID-19 leve.

## INTRODUCTION

In the last quarter of 2019, the coronavirus disease or severe acute respiratory syndrome corona virus 2 (SARS-CoV-2) outbreak occurred in China. Subsequently, it has spread rapidly worldwide. The symptoms of coronavirus disease (COVID-19) range from mild flu to severe pneumonia, often presenting with fever, cough, sore throat, shortness of breath, and fatigue^(1)^.

Retinal vein occlusion (RVO) is a multifactorial vascular disease characterized by retinal blood stasis, increased venous tortuosity, intraretinal hemorrhage, and macular edema, which may cause loss of vision or blindness. In addition to systemic vascular diseases such as hypertension, arteriosclerosis, and diabetes mellitus, genetic predisposition and environmental factors have been reported to be associated with the risk of RVO^(2,3)^. The most commonly attributed reason for the development of RVO in COVID-19 is “sepsis-induced coagulopathy”^(4)^.

Herein, we present a case of bilateral branch RVO (BRVO) that occurred sequentially months after the onset of the COVID-19 disease.

## CASE REPORT

A 48-year-old male patient was admitted to our hospital with symptoms of possible mild COVID-19 and was diagnosed as having SARS-CoV-2 infection after polymerase chain reaction testing. Chest computed tomography findings indicated COVID-19. Strict follow-up with outpatient medical treatment and home isolation was applied to the patient. As the disease stage was moderate, the patient did not experience oxygen starvation.

He received therapy with a combination of low-molecular-weight heparin (enoxaparine), pantoprazole, and favipiravir (antiviral). He recovered in the first week after treatment and was recommended to continue quarantine for an additional 1 week.

Two months after the diagnosis of COVID-19, he presented with decreased and blurred vision in his left eye. His familial and retinal or systemic disease histories were unremarkable.

**Figure 1:**
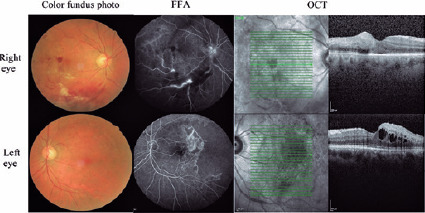
Dilatation and tortuosity of the affected venous segment, with flame-shaped hemorrhages, cotton wool spots in the affected section of the retina drained by the obstructed vein in both eyes, ischemia, and arteriovenous shunt at initial presentation.

On ophthalmologic assessment, his best-corrected visual acuities were 100/100 and 20/100 in the right and left eyes, respectively. No significant findings were obtained in the anterior segment examinations of both eyes. The intraocular pressure measurement using the Goldmann applanation tonometer was within the normal limits. The D-dimer level was well above normal in the coagulation profile. Treatment was started for BRVO and the coagulation. In the third month of the BRVO therapy, the same symptoms developed in the other eye (right), and the visual acuity decreased to 10/100, and subsequently, the eye was diagnosed with BRVO. Figure 1 shows dilatation and tortuosity of the affected venous segment, with flame-shaped hemorrhages, retinal edema, and cotton wool spots drained by the obstructed vein in both eyes. Optical coherence tomography (OCT) revealed serous macular detachment and retinal thickening in both eyes. Fundus fluorescein angiography (FFA) exhibited a capillary filling defect and leakage and aneurysms, which were observed in the affected retina in both eyes (Figure 1).

**Figure 2:**
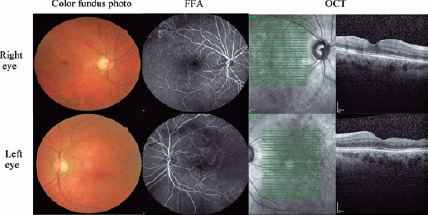
Arteriovenous shunt, ischemia, and ghost vessels in both eyes after treatment.

Ghost vessels with venous folds were found in the affected retina in both eyes. OCT revealed retinal thinning with a cystoid pattern in the temporal macula of both eyes. FFA analysis revealed a capillary filling defect and leakage with arteriovenous (A-V) shunt and ischemia in the temporal macula of both eyes (Figure 2). Finally, the visual acuity increased to 40/100 in the right eye and 30/100 in the left eye after treatment.

## DISCUSSION

Recent studies have shown that patients with a mild-to-severe COVID-19 diagnosis may develop A-V thromboembolism after recovery^(5,6)^. Two causes are responsible for the vascular damage in patients with COVID-19. One is the hypercoagulable state called diffuse intravascular coagulation (DIC)-like condition, and the other is the vasculitic process of the endothelial cell directly related to viral infection and the resulting widespread endothelial inflammation^(7,8)^.

Gaba et al. reported that a patient diagnosed as having COVID-19 pneumonia with hypertension and morbid obesity can develop an inflammatory condition resulting in bilateral central RVO^(9)^. On the contrary, in our case, the disease stage was mild, and bilateral BRVO developed at different times points after recovery from the disease.

Higher D-dimer levels were detected in patients with COVID-19 by an almost tenfold increment in comparison with the interleukin-6 levels, which indicates a thrombotic activity after stimulation of cellular activation possibly induced by the virus. Recent studies have demonstrated that high D-dimer levels showed a strong correlation with disease severity and prognosis in the development of thrombotic complications of COVID-19, such as pulmonary embolism, stroke, and DIC, even with hyperbaric O^2^ therapy^(10,11,12)^. In addition to these studies, the D-dimer level may be a prognostic marker of RVO in patients with COVID-19 infection.

We report this interesting case of BRVO that developed months after the diagnosis of mild COVID-19 infection, apart from other etiological factors known to date. Physicians should be vigilant that such complications may develop months after treatment in patients with a history of COVID-19.
